# Comprehensive characterization of m6A methylation and its impact on prognosis, genome instability, and tumor microenvironment in hepatocellular carcinoma

**DOI:** 10.1186/s12920-022-01207-x

**Published:** 2022-03-08

**Authors:** Tengfei Yin, Lang Zhao, Shukun Yao

**Affiliations:** 1 Peking University China-Japan Friendship School of Clinical Medicine, No. 2 Yinghua East Road, Chaoyang District, Beijing, 100029 China; 2 Department of Gastroenterology, China-Japan Friendship Hospital, Beijing, China; 3 Peking Union Medical College and Chinese Academy of Medical Sciences, Beijing, China

**Keywords:** M6A modification, Genome instability, Tumor microenvironment, Hepatocellular carcinoma

## Abstract

**Background:**

N6-methyladenosine (m6A) RNA regulation was recently reported to be important in carcinogenesis and cancer development. However, the characteristics of m6A modification and its correlations with clinical features, genome instability, tumor microenvironments (TMEs), and immunotherapy responses in hepatocellular carcinoma (HCC) have not been fully explored.

**Methods:**

We systematically analyzed the m6A regulator-based expression patterns of 486 patients with HCC from The Cancer Genome Atlas and Gene Expression Omnibus databases, and correlated these patterns with clinical outcomes, somatic mutations, TME cell infiltration, and immunotherapy responses. The m6A score was developed by principal component analysis to evaluate m6A modifications in individual patients.

**Results:**

M6A regulators were dysregulated in HCC samples, among which 18 m6A regulators were identified as risk factors for prognosis. Three m6A regulator-based expression patterns, namely m6A clusters, were determined among HCC patients by m6A regulators with different m6A scores, somatic mutation counts, and specific TME features. Additionally, three distinct m6A regulator-associated gene-based expression patterns were also identified based on prognosis-associated genes that were differentially expressed among the three m6A clusters, showing similar properties as the m6A regulator-based expression patterns. Higher m6A scores were correlated with older age, advanced stages, lower overall survival, higher somatic mutation counts, elevated PD-L1 expression levels, and poorer responses to immune checkpoint inhibitors. The m6A score was validated as an independent and valuable prognostic factor for HCC.

**Conclusion:**

M6A modification is correlated with genome instability and TME in HCC. Evaluating m6A regulator-based expression patterns and the m6A score of individual tumors may help identify candidate biomarkers for prognosis prediction and immunotherapeutic strategy selection.

**Supplementary Information:**

The online version contains supplementary material available at 10.1186/s12920-022-01207-x.

## Background

Hepatocellular carcinoma (HCC) accounts for the most cases of primary liver cancers, which have the sixth and third highest incidence and mortality rate, respectively [1]. HCC is characterized by its high malignancy, high recurrence rate, and poor prognosis. However, its underlying molecular pathogenesis remains largely unclear.

N6-methyladenosine (m6A) RNA methylation plays a vital role in RNA splicing, export, processing, translation and decay, and is the most common type of post-transcriptional regulation of mRNAs in living organisms [2]. Previous reports identified the following three types of m6A regulators: “writers” (methyltransferases), “readers” (m6A-binding proteins), and “erasers” (demethylases) [3]. Recently, growing evidence has revealed the emerging role of m6A deregulation in liver diseases and cancer [4–6]. Thus, regulators of m6A modification may be diagnostic and therapeutic targets for HCC.

Genome instability is an evolving hallmark of many types of cancers and is involved in tumorigenesis and cancer progression [7]. The tumor mutation burden (TMB), which refers to the accumulation of endogenous and exogenous mutation processes in cancer cells, has been reported to play a crucial role in the biological processes of cancer based on the high frequency and wide spectrum of somatic mutations [8]. The TMB was also shown to be a useful prognostic biomarker [9], and for immunotherapy selection in some cancers [10]. Furthermore, Zhang et al*.* found that m6A modification scores were correlated with TMB levels in gastric cancer [11]. However, the relationship between m6A regulation and genome instability in HCC remains ambiguous.

Cancer development has been found to occur that are closely associated with alterations in tumor microenvironments (TMEs), which are aggregations of tumor cells and adjacent tumor-related nontumor cells [12]. Blockade of immune checkpoints, including programmed cell death-1 (PD-1), its ligand (PD-L1), and cytotoxic T lymphocyte associated antigen 4 (CTLA4), has revolutionized oncology therapeutics [13, 14]. The association between PD-L1 expression and immune infiltration of m6A regulators has been reported in head and neck squamous cell carcinoma and gliomas [15, 16]. However, the relationships of m6A modification regulators with immune infiltration of TMEs and immunotherapy in HCC require further exploration.

The aim of this study was to comprehensively identify m6A regulator-based expression patterns, quantify individual scores, and evaluate the prognostic value of m6A methylation in HCC. The relationships of m6A regulation with genome instability and TMEs were also investigated to reveal the role of m6A methylation in HCC carcinogenesis and immunotherapy. In summary, the current study expanded the understanding on the mechanism of m6A modification in the tumorigenesis and development of HCC and identified biomarker candidates for prognosis prediction and therapy selection in patients with HCC.

## Methods

### Data collection

The RNA profiles and clinical information of 374 HCC samples and 50 normal samples were downloaded from The Cancer Genome Atlas (TCGA) database on July 18, 2021. The fragments per kilobase of transcript per million mapped reads (FPKM) values of RNA expression data downloaded from TCGA database were transformed into transcripts per kilobase million (TPM) values. A total of 21 datasets of HCC patients were obtained from Gene Expression Omnibus (GEO) databases. After excluding 20 GEO cohorts without survival data, the normalized series matrix file and clinical information of 115 HCC patients were downloaded from the GSE76427 dataset. Next, 3 samples were deleted from TCGA cohort because they were not primary tumors of HCC. Therefore, the gene expression and clinical data of 486 patients with HCC were further analyzed. The batch effects among TCGA and GSE76427 cohorts were removed using the ComBat method in “sva” R package. The PRISMA flow chart is shown in Additional file 1: Fig. S1. The somatic mutation data of patients with HCC were obtained from TCGA database and identified using the VarScan software. The copy number variation (CNV) data of HCC were collected from the UCSC Xena database (www.xena.ucsc.edu). The immunophenoscores (IPS), a predictor of response to anti-CTLA4 and anti-PD-1 antibodies based upon the expression profiles of the representative genes of immunomodulatory, were obtained from The Cancer Immunome Atlas (https://tcia.at). We followed the rules that are set forth by the public data set management for use of the data set. All data in this study were obtained from public databases and available to the public.

### Expression variation and prognostic value of m6A regulators in HCC

According to published studies, 25 m6A methylation regulators were examined, including 10 writers (*METTL3/14/16*, *WTAP*, *VIRMA*, *ZC3H13*, *CBLL1*, *ZCCHC4*, *RBM15* and *RBM15B*), 13 readers (*YTHDC1/2*, *YTHDF1/2/3*, *IGF2BP1/2/3*, *HNRNPC*, *FMR1*, *LRPPRC*, *ELAVL1* and *HNRNPA2B1*), and 2 erasers (*FTO* and *ALKBH5*) [11, 17–20]. The mRNA expression levels of m6A regulators were compared between the normal and HCC samples. The somatic mutation counts and CNV alterations of the 25 m6A regulators are summarized and illustrated. Survival analysis was utilized to screen prognosis-related m6A regulators.

### Consensus clustering of m6A regulator-based expression patterns

Based on the expression levels of m6A regulators, patients with HCC in TCGA and GSE76427 cohorts were classified into different m6A regulator-based expression patterns, namely m6A clusters, using the “ConsensusClusterPlus” R package with the pam method, with 50 iterations and a resampling rate of 80% [21]. To determine the number of m6A clusters, we used the empirical cumulative distribution function (CDF) plots to identify the consensus distributions for each k, as well as the delta area score to display the relative growth in cluster stability. Consistent matrix (CM) plots were also illustrated based on the k-value. Moreover, based on the expression level of 25 m6A regulators, principal component analysis (PCA) was performed among the different m6A clusters. The expression levels of the 25 m6A regulators were compared among the m6A regulator-based expression patterns.

### Enrichment analysis and immune cell infiltration of m6A clusters

To explore the different biological processes between m6A clusters, the “c2.cp.kegg.v7.4.symbols” file was downloaded from the MsigDB database for Gene set variation analysis (GSVA). An adjusted *P* < 0.05 was considered to indicate statistically significant results. The “limma” R package was applied to identify differentially expressed and m6A-related genes using the lmFit and eBayes functions with the significant cutoff value at adjusted *P* < 0.05. Differentially expressed genes (DEGs) between the different m6A regulator-based expression patterns were first identified, and then the Kyoto Encyclopedia of Genes and Genomes (KEGG) analysis was conducted based upon the DEGs by using the “clusterProfiler” R package [22, 23]. Furthermore, the immune infiltration characteristics were analyzed and compared among different m6A clusters. The relative abundances of cell infiltration in the TMEs of HCCs were quantified using the enrichment score for each immune category of single-sample gene set enrichment analysis (ssGSEA). The “GSVA” R package was utilized for GSVA and ssGSEA analyses.

### Identification of m6A gene clusters

To further analyze the values of the DEGs of the m6A clusters, univariate Cox regression analysis was performed to retrieve the prognosis-related DEGs (*P* < 0.0001). HCC patients in TCGA and GEO cohorts were separated into different m6A regulator-associated gene-based expression patterns, namely m6A gene clusters, which were defined by the consensus clustering according to the expression of prognostic DEGs. PCA of m6A gene clusters was performed based on expression of these prognosis-related DEGs. Survival analysis was also conducted among gene clusters. Moreover, the expression levels of the 25 m6A regulators in the different gene expression-based clusters were then investigated.

### Correlation of m6A score with clinical factors, genome stability, and immune characteristics

To quantify the m6A modification score, PCA was conducted to develop a set of m6A scoring systems using a method similar to that used by Zhang et al*.* [11]. Briefly, the sum of all principal components 1 and 2 of each prognostic DEG related to m6A regulators was considered as the m6A score. Patients with HCC were classified into the low or high m6A score subgroup according to the cutoff point determined using “survminer” R package. The differences in m6A scores among the m6A clusters and m6A gene clusters were compared. Furthermore, the value of m6A score in predicting prognosis was examined by survival analysis of patients with HCC and of patients in subgroups stratified by clinicopathological features. The correlations of the m6A score with the TMB, immune cell infiltration and IPS were explored in detail to reveal the roles of m6A regulators.

### Statistical analysis

R version 4.0.3 was utilized for data analysis. For quantitative data, *t*-test and Wilcoxon test were used to compare two groups, whereas one-way analysis of variance and Kruskal–Wallis test were used to compare differences among three or more groups for parametric and nonparametric data, respectively. Kaplan–Meier curves were used for survival analyses using log-rank test. The cutoff values of m6A regulator expression and the m6A score in survival analyses were calculated using the surv-cutpoint function in “survminer” R package to evaluate all potential cutoff points repeatedly to determine the maximum rank statistics. Cox regression analyses were performed to identify independent prognostic factors from the m6A regulators and m6A-related prognostic DEGs. Time-dependent receiver operating characteristic (ROC) curves were constructed to evaluate the accuracy of the m6A score for prognosis prediction using the 1- and 3-year areas under the curves (AUCs). Waterfall maps were used to present the mutational landscape of patients with HCC in TCGA cohort using the “Maftools” R package. Statistical significance was set at *P* < 0.05.

## Results

### Mutational genomic landscape of m6A regulators in HCC

**Fig. 1:**
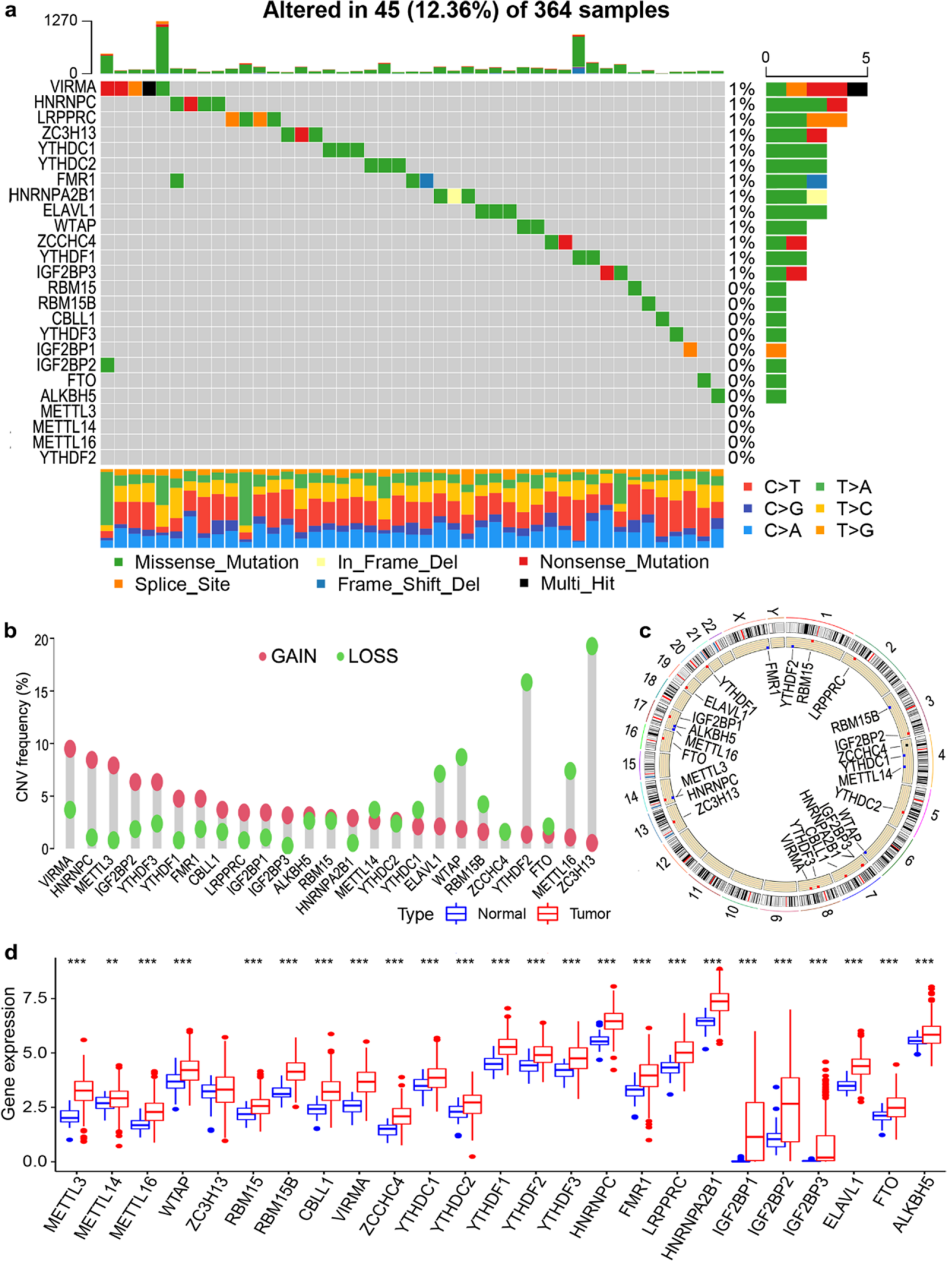
Genetic variation landscape of m6A regulators for patients with hepatocellular carcinoma (HCC) in TCGA cohort. **a** Mutation frequency in 25 m6A regulators. **b** Copy number variation (CNV) frequency in 25 m6A regulators. **c** Chromosomal locations of the CNV alterations in the m6A regulators. **d** Differences in expression levels of the 25 m6A regulators between HCC and normal samples. *P < 0.05; **P < 0.01; ***P < 0.001

A total of 364 patients with HCC were enrolled from TCGA cohort to depict the landscape of genetic variation in m6A regulators, among which 45 samples (12.36%) showed mutations in m6A regulators (Fig. 1a). The CNV alteration of HCC is depicted in Fig. 1b. Some m6A regulators displayed increased copy numbers, whereas others showed decreased CNV frequencies. The chromosome locations of the CNV alterations of m6A regulators are illustrated in Fig. 1c. Furthermore, the expression levels of m6A regulators were compared between normal and HCC tissues in TCGA cohort, indicating that except for *ZC3H13*, the other 24 m6A regulators were upregulated in the HCC samples in TCGA cohort (Fig. 1d).

### Prognostic value of m6A regulators in HCC

**Fig. 2:**
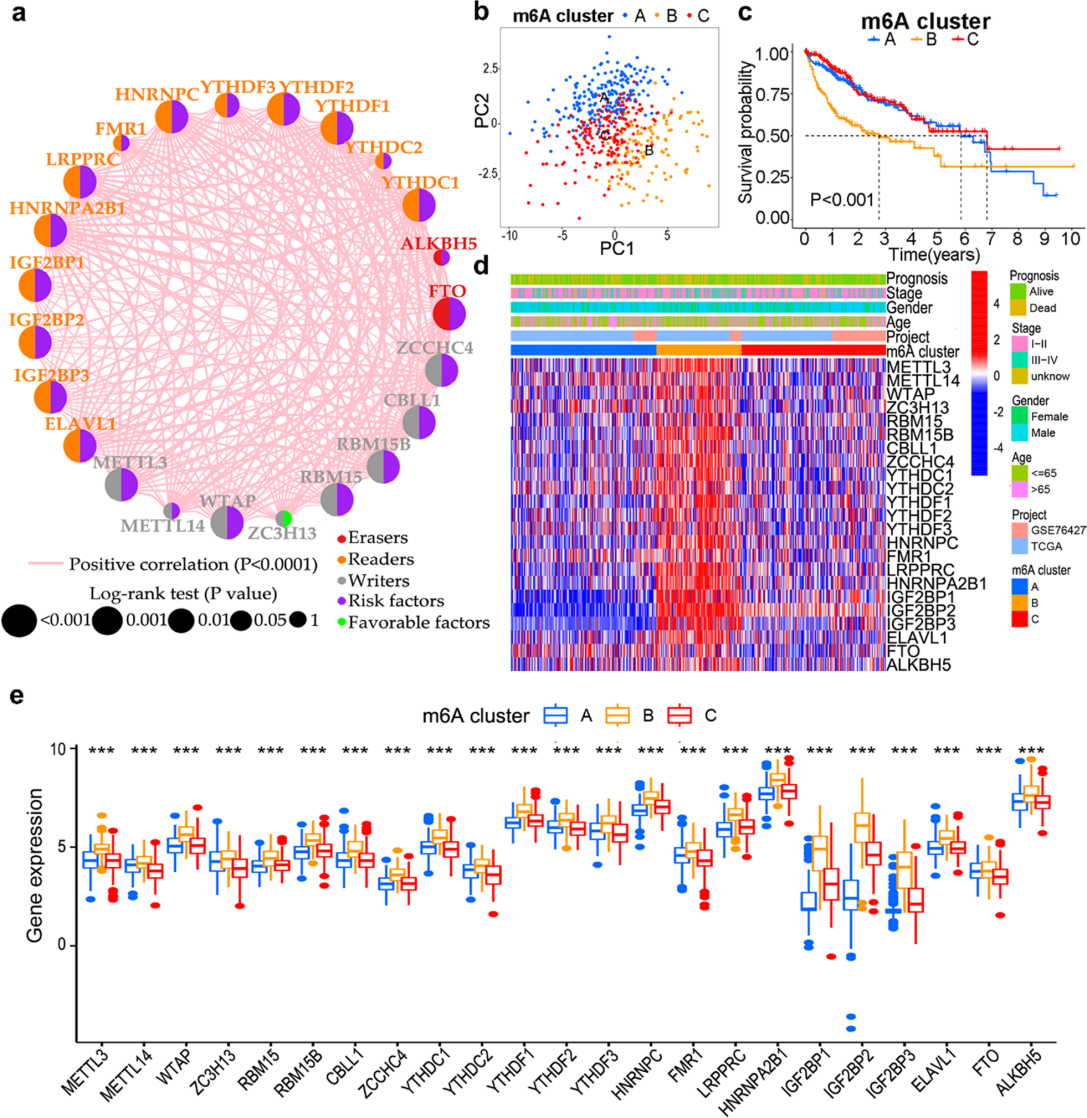
Three m6A regulator-based expression patterns in patients with HCC. **a** Network generated by R software to show interactions and prognostic effects of m6A regulators. The lines linking regulators represent Pearson’s correlations, and the thickness of the line represents the correlation strength. The size of each circle represents the prognostic effect of each regulator and scaled by *P* calculated by log-rank test. Purple dots in the circle represent risk factors of prognosis, whereas green dots represent protective factors. **b** Principal component analysis plot showing the transcriptomic data among three m6A clusters. **c** Survival analysis of m6A clusters. **d** Clinicopathological features and expression levels of m6A regulators in m6A clusters. **e** Expression levels of m6A regulators in m6A clusters

As *METTL16* and *VIRMA* were not available in the GSE76427 cohort, only 23 m6A regulators were analyzed. A total of 18 m6A regulators, namely, *METTL3, WTAP, RBM15, RBM15B, CBLL1, ZCCHC4, YTHDC1, YTHDF1/2/3, LRPPRC, HNRNPA2B1, HNRNPC, IGF2BP1/2/3, ELAVL1*, and *FTO*, were found to be associated with overall survival (OS) in HCC patients from TCGA and GSE76427 cohorts using Kaplan–Meier curves and log-rank tests (Additional file 1: Fig. S2). A crosstalk network was generated by R software to present the widespread correlations in expression levels across the m6A regulators in patients with HCC (Fig. 2a). In summary, m6A regulators could have played a vital role in predicting clinical outcomes in patients with HCC, and some regulators showed potential as prognostic biomarkers.

### Consensus clustering for HCC patients by m6A regulators

Using consensus clustering, the patients with HCC from TCGA and GSE76427 cohorts were assigned to three m6A regulator-based expression patterns, namely m6A cluster A (n = 188), cluster B (n = 110), and cluster C (n = 187), based on the expression of m6A regulators. Both the CDF plot and delta area score showed the highest stability with k = 3 (Additional file 1: Fig. S3a, b). The CM plot also showed the high consistency at k = 3 (Additional file 1: Fig. S3c). The results of PCA showed distinguishable differences in the transcriptional profiles among the three m6A clusters (Fig. 2b). The three m6A clusters showed significantly different OS, with cluster A and C exhibiting good prognoses and cluster B demonstrating the worst clinical outcome (Fig. 2c). The relationships of the m6A clusters with clinicopathological features and expression of m6A regulators are illustrated in Fig. 2d. Furthermore, the expression of the m6A regulators among the three m6A clusters showed significant differences (Fig. 2e). Specifically, cluster B was characterized by significant upregulation of all m6A regulators.

### Enrichment analysis and TMEs of m6A clusters

**Fig. 3:**
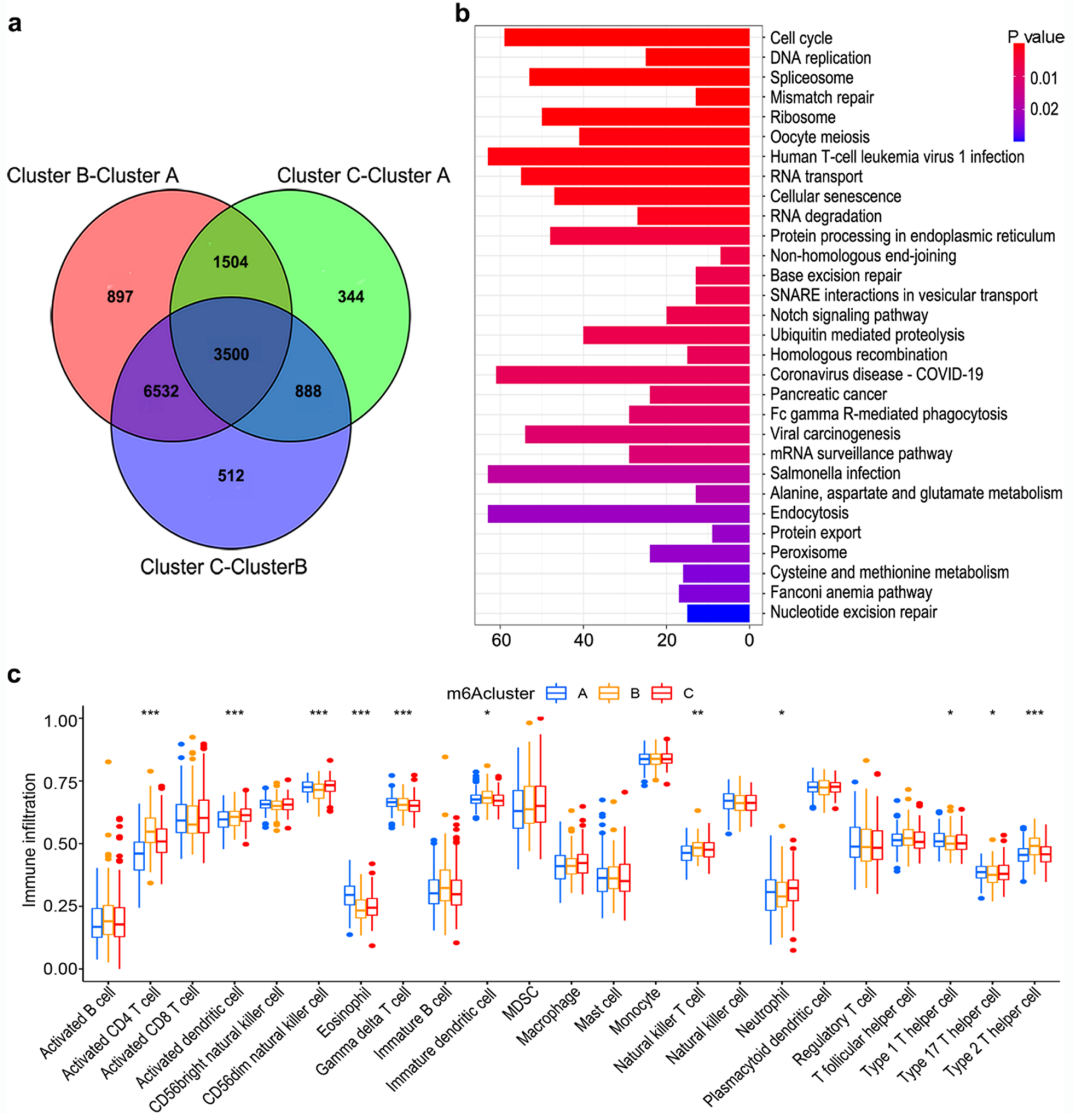
Enrichment analysis and immune cell infiltration in m6A clusters. **a** Differentially expressed genes (DEGs) between the different m6A clusters. **b** Functional annotation of the DEGs using KEGG analysis. **c** Immune cell infiltration in three m6A clusters

GSVA analyses suggested that among the three m6A clusters, cluster B included carcinogenesis-related enrichment pathways such as cell cycle, RNA degradation and nucleotide excision repair (Additional file 1: Fig. S4a, S4b). A total of 3500 genes were found to be differentially expressed among the three m6A clusters (Fig. 3a). KEGG enrichment analysis suggested that these DEGs were enriched in carcinogenesis and RNA modification pathways, including cell cycle, mismatch repair, RNA degradation, and nucleotide excision repair (Fig. 3b). The cell infiltration of the TMEs in m6A clusters was further investigated. Cluster B showed the highest infiltration of activated CD4^+^ T cells, immature dendritic cells, natural killer T cells, and type 2 T helper cells, with the lowest infiltration of eosinophils, neutrophils, type 1 T helper cells, and type 17 T helper cells (Fig. 3c). Thus, the immunosuppressive TMEs in cluster B may have contributed to worse clinical outcomes, indicating that the distinct TME characteristics in varying m6A clusters might play a vital role in the tumorigenesis and prognosis of HCC.

### Consensus clustering of the m6A gene cluster

**Fig. 4:**
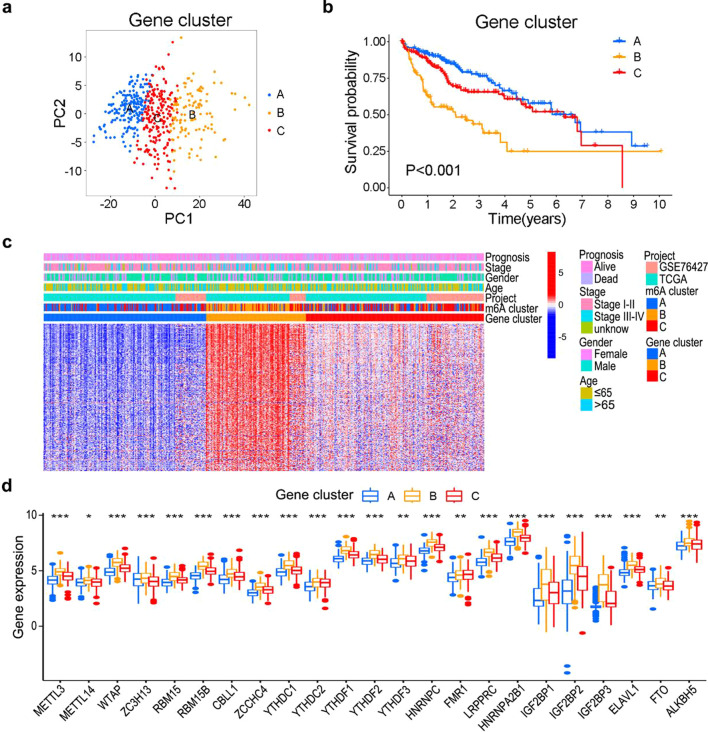
Three m6A gene clusters of patients with HCC. **a** Principal component analysis (PCA) of the three m6A gene clusters. **b** Survival analysis of m6A gene clusters. **c** Clinicopathological features and expression levels of DEGs in the m6A gene clusters. **d** Differences in expression levels of m6A regulators among the three m6A gene clusters

Using the univariate Cox regression model, 307 DEGs were extracted as significantly associated with prognosis (Additional file 1: Table S1). Based on the expression of these prognostic DEGs, patients with HCC were classified into three m6A regulator-associated gene-based expression patterns: m6A gene cluster A (n = 179), gene cluster B (n = 110), and gene cluster C (n = 196) (Additional file 1: Fig. S3d–f). Distinguishable differences in the transcriptional profiles of prognostic DEGs were revealed among the three m6A gene clusters (Fig. 4a). Consistent with the results for the three m6A clusters mentioned above, the OS of the three gene clusters were significantly different, with cluster B exhibiting the worst prognosis (Fig. 4b). The relationships of the m6A gene cluster with age, gender, stage, prognosis, m6A cluster, and expression levels of DEGs are illustrated in Fig. 4c. Furthermore, the expression levels of the 23 m6A regulators differed among the three gene clusters, with gene cluster B exhibiting the highest expression of most m6A regulators (Fig. 4d). In summary, the m6A-related DEGs may be crucial factors affecting the clinical outcomes of patients with HCC.

### Generation of m6A score and the prognosis prediction

**Fig. 5:**
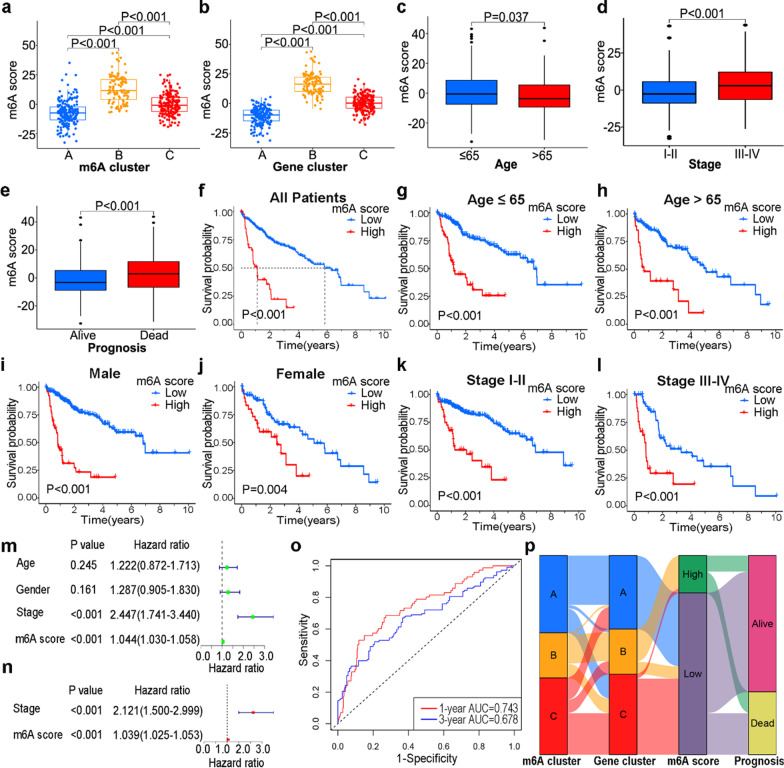
Development of m6A score. Differences in m6A scores among m6A clusters (**a**) and m6A gene clusters (**b**). M6A scores of patients at different ages (**c**), stages (**d**), and prognoses (**e**). **f** Survival analysis of the m6A score in all patients with HCC. Survival analyses of patients in different clinical subgroups, including ≤ 65 years (**g**), > 65 years (**h**), male (**i**), female (**j**), stage I–II (**k**), and stage III–IV (**l**). Univariate Cox regression analysis (**m**) and multivariate Cox analysis (**n**) of m6A scores in patients with HCC. **o** Receiver operating characteristic curves and area under the curve (AUC) of the m6A score for prognostic prediction. **p** Alluvial diagram of changes in m6A clusters, gene clusters, m6A scores and prognosis

To further explore the value of the DEGs, an m6A score was generated to evaluate the m6A modification. The m6A score was highest in cluster B and lowest in cluster A (Fig. 5a). Similar distribution was observed in the m6A gene clusters (Fig. 5b). Older patients (age > 65 years), patients with early-stage disease and patients survived at the clinical endpoint had lower m6A scores compared to their control pairs (Fig. 5c–e). Using a cutoff value of 10.46 determined by the “survminer” R package, the patients were divided into high and low m6A score groups. Patients with low m6A scores tended to have better prognoses (Fig. 5f). The OS of the low m6A score group was longer than the high m6A score group of HCC patients, suggesting that the m6A score can be successfully applied to all patients with HCC as a prognostic indicator (Fig. 5g–l). The age, gender, stage and m6A score were examined using Cox regression analysis in HCC patients. Univariate Cox analysis showed that the stage and m6A score (both *P* < 0.001) were associated with OS of HCC patients (Fig. 5m). The stage and m6A score (both *P* < 0.001) were also correlated with OS in multivariate Cox analysis (Fig. 5n). Therefore, the m6A score was validated as an independent prognostic factor for patients with HCC. The 1- and 3-year AUC of the m6A score were 0.743 and 0.678 respectively, indicating good performance of the m6A score in predicting prognoses (Fig. 5o). An alluvial diagram was utilized to illustrate the attribute changes among m6A clusters, m6A gene clusters, m6A scores, and clinical outcomes (Fig. 5p).

### Correlation of m6A score with genomic instability

**Fig. 6:**
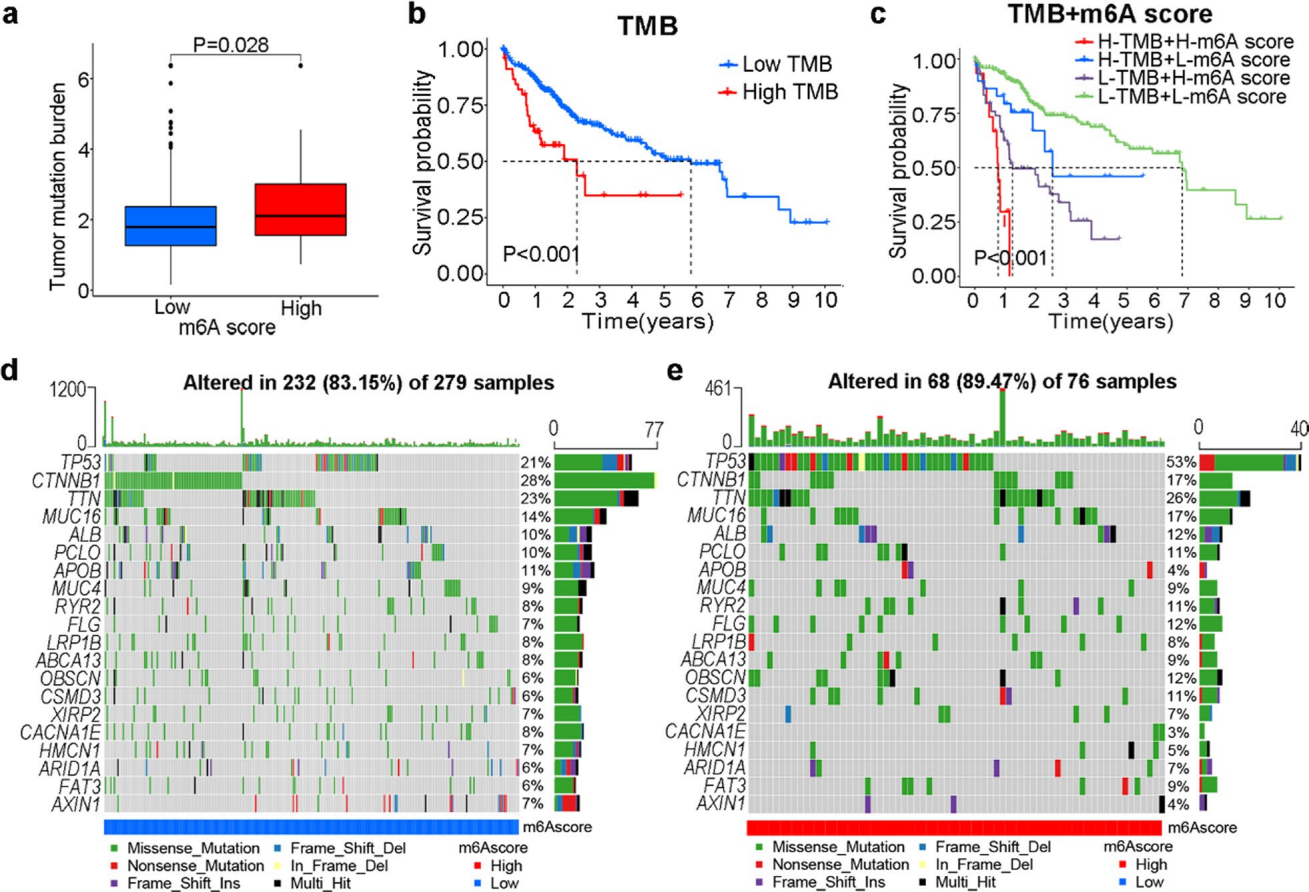
Correlation of m6A score with tumor mutation burden (TMB). **a** TMB levels in different m6A score groups. **b** Survival analysis of TMB in patients with hepatocellular carcinoma. **c** Survival analysis of patient subgroups stratified according to m6A score and TMB. Waterfall maps of somatic mutations for patients with low m6A scores (**d**) and high m6A scores (**e**). H: high; L: low

The somatic mutation count of the high m6A score subtype was higher than that of the low score subtype (Fig. 6a). Patients with high TMBs tended to have poorer prognoses (Fig. 6b). The m6A score was combined with the TMB to predict the prognoses of patients with HCC (Fig. 6c). Specifically, patients with an elevated TMB and m6A scores showed the worst prognoses. Waterfall maps of low and high m6A score groups revealed the distribution of somatic mutations in the TCGA cohort (Fig. 6d, e). The top three mutated genes in the low m6A score group were *CTNNB1* (28%), *TTN* (23%) and *TP53* (21%), and the top three mutated genes were *TP53* (53%), *TTN* (26%) and *CTNNB1* (17%) in the high m6A score group. Based on these results, m6A modification may be correlated with genomic stability, but the mechanism requires further exploration.

### Relationship of m6A score with TME and immunotherapy

**Fig. 7:**
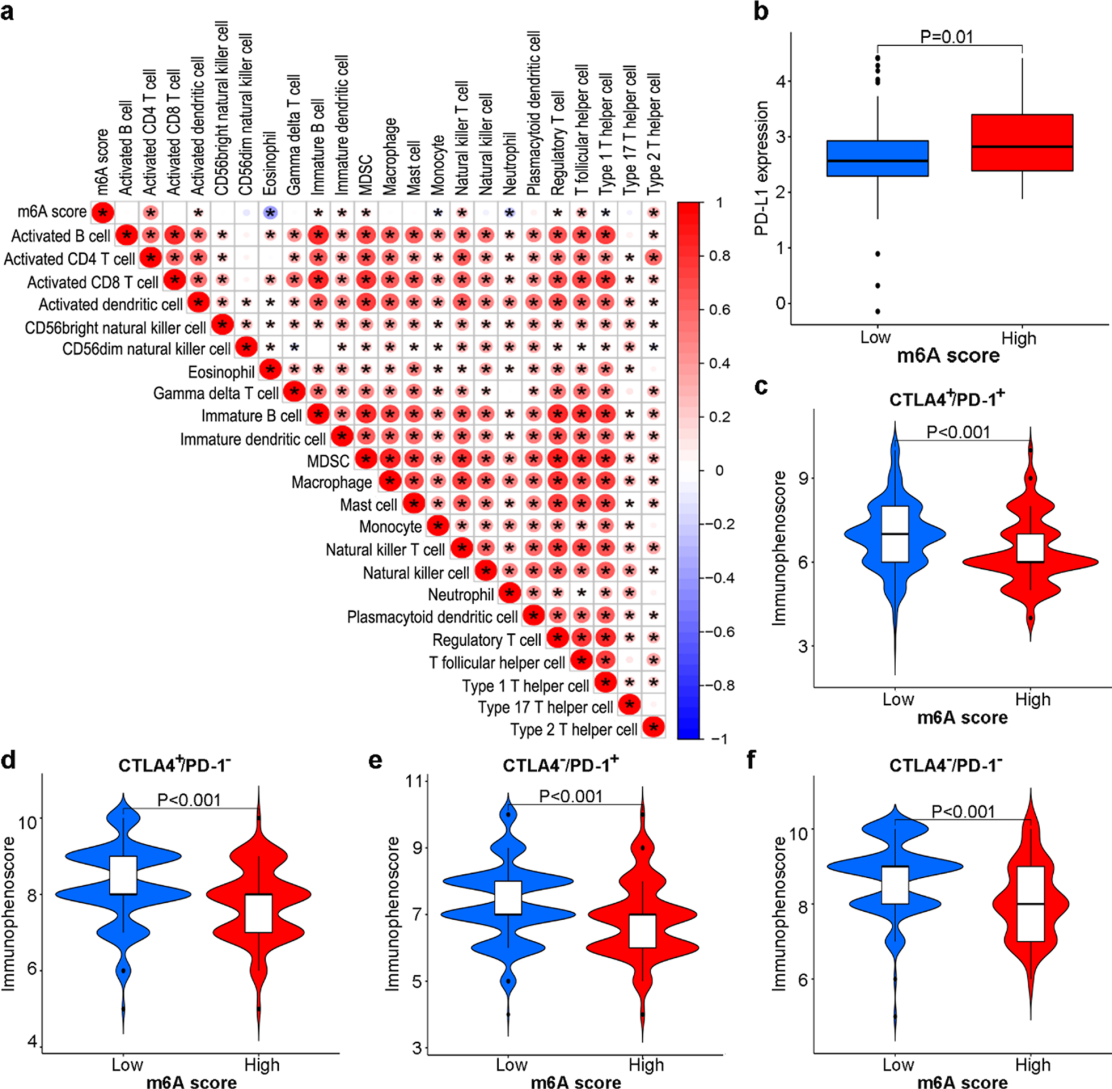
Relationship of m6A scores with tumor microenvironments. **a** Correlation of the m6A score with immune cell infiltration. **b** PD-L1 expression in groups with low and high m6A scores. The immunophenoscore of different m6A score groups in patients with CTLA4^+^/PD-1^+^ (**c**), CTLA4^+^/PD-1^−^ (**d**), CTLA4^−^/PD-1^+^ (**e**), and CTLA4^−^/PD-1^−^ (**f**)

The m6A scores were positively correlated with activated CD4^+^ T cells, activated dendritic cells, immature B cells, immature dendritic cells, myeloid-derived suppressor cells (MDSCs), natural killer T cells, regulatory T cells, T follicular helper cells and type 2 T helper cells, and negatively correlated with eosinophils, monocytes, neutrophils, and type 1 T helper cells (Fig. 7a). Patients with high m6A scores were characterized by increased expression levels of PD-L1 (Fig. 7b). As low m6A scores were associated with a higher IPS in the four subgroups, patients with low m6A scores showed better responses to anti-PD-1 therapy and anti-CTLA4 therapy (Fig. 7c–f). Overall, m6A modification might be involved in immune cell infiltration of the TME and act as a promising crucial factor in the immunotherapy response.

## Discussion

Mounting evidence suggests that m6A methylation is a prevalent RNA internal modification with an essential role in HCC carcinogenesis, progression, and treatment outcomes [5, 6]. The correlation between m6A regulation and the TME has been investigated in some cancer types such as gastric cancer [11] and lung adenocarcinoma [17]. In this study, we identified three m6A regulator-based expression patterns and three m6A gene clusters and developed an m6A score to quantify m6A modification. The relationships of m6A regulation with clinical outcomes, somatic mutations, cell infiltration of the TME, and immunotherapy responses were systematically investigated to examine the value of m6A modification in HCC development.

Distinct genetic alterations and significantly upregulated expression of m6A regulators were observed in patients with HCC compared to in normal pairs in this study, which is consistent with results obtained in other cancer types, such as gastric cancer, colorectal cancer, and pancreatic cancer [11, 19, 24]. The m6A regulators were rarely mutated in HCC, whereas gain and loss alterations in CNVs were prevalent but relatively equal among the m6A regulators. These results indicated that somatic mutations and CNVs may partially explain the expression differences in m6A regulators between HCC and normal samples, which requires further exploration.

In our study, 18 m6A regulators were identified as prognostic indicators for HCC. High expression of these m6A regulators was correlated with worse OS in patients with HCC. Consistent with our findings, *METTL3* [25], *WTAP* [6], *ZCCHC4* [26], *YTHDF2* [5], *LRPPRC* [27], *IGF2BP1* [28], *IGF2BP2* [29], and *FTO* [30] have been reported as potential prognostic indicators involved in diverse pathophysiological processes in HCC. Moreover, higher m6A scores correlated with older age, advanced stage, higher somatic mutation counts, and poorer OS in all age, gender, and stage subgroups with good performance in our study. Meanwhile, m6A scores were found to be useful for diagnostic and prognostic prediction in gastric cancer [11], colon cancer [19], and HCC [31]. In summary, m6A regulators and the m6A score may be promising biomarkers for evaluating clinicopathological features and predicting clinical outcomes in HCC.

Our findings also revealed a relationship between m6A regulation and the TME in HCC. Patients with higher m6A scores showed elevated PD-L1 expression. Accumulating evidence has shown that m6A regulation is correlated with PD-L1 expression and TMEs in gastric cancer [11] and colorectal cancer [19]. In addition, the m6A regulator-related risk scores were previously found to be correlated with CTLA4 and PD-L1 in breast cancer [32]. Analysis of the value of m6A modification in immunotherapeutic clinical outcomes of HCC suggested that patients with low m6A scores might benefit from immunotherapies targeting CTLA4/PD-1 inhibitors. Thus, m6A regulators might affect PD-L1 expression and immune cell infiltration in patients with HCC.

In our study, TMB could act as a potential prognostic factor for HCC, which is consistent with many other studies [33, 34]. Our findings revealed that combination of m6A score and TMB could predict OS of HCC. Moreover, *TP53* was the most common mutation (53%) in patients with high m6A scores, whereas *CTNNB1* was the most common mutation (28%) in patients with low m6A scores. Recent studies indicated that *TP53* mutations are correlated with the TMEs in HCC [35], and HCC patients harboring *TP53* mutations tended to have poor prognosis along with hypoxia-induced HCC stemness [36]. Patients with HCC carrying TP53 neoantigens also showed longer OS and higher cytotoxic lymphocyte infiltration [37]. Inhibiting the expression of *CTNNB1* may increase the stemness features of HCC [38]. Overall, the correlation and interaction of m6A modifications with genome instability might influence tumorigenesis and prognosis of HCC.

In this study, we provided new insight into the roles of m6A regulator-based expression patterns in HCC. In clinical practice, the m6A score may be useful for evaluating m6A methylation, predicting clinical outcome, and assessing corresponding TME cell infiltration characterization, TMB and immunotherapeutic response in individual patients with HCC, which might contribute to the identification of prognostic biomarkers and selection of immunotherapies. However, there were some limitations to our study. First, the sample size of patients may affect the development of the m6A score. Large-scale datasets and clinical samples should be utilized to validate the prognostic value of the m6A score and m6A regulators found in this study. Moreover, molecular experiments are necessary to confirm the specific biological pathways involved in m6A modification during HCC carcinogenesis and progression. Finally, considering the limitations in the therapeutic response data, the efficiency of m6A modification to predict prognosis and clinical benefit in immunotherapy requires verification in the future.

## Conclusions

In summary, we identified m6A regulator-based expression patterns and m6A score, and investigated their prognostic value in HCC patients. Furthermore, the correlation of m6A regulation with somatic mutations, TMEs, and immunotherapy responses were explored. Our findings provide insights into the evaluation of m6A regulation as a potential biomarker for prognostic prediction and guidance of immunotherapeutic strategies for HCC.

## Supplementary Information

**Additional file 1.** **Fig. S1.** PRISMA flow diagram for data collection. **Fig. S2.** Survival analyses for m6A regulators associated with overall survival of patients with HCC. **Fig. S3.** Unsupervised consensus clustering analysis of patients with HCC. **Fig. S4.** GSVA analyses of the three m6A regulator-based expression patterns. **Table S1.** Prognostic m6A-related genes among three m6A regulator-based expression patterns.

## Data Availability

Publicly available datasets were analyzed in this study. The data analyzed in this study are available on The Cancer Genome Atlas database (https://portal.gdc.cancer.gov/), Gene Expression Omnibus database (https://www.ncbi.nlm.nih.gov/geo/), UCSC Xena database (www.xena.ucsc.edu), and The Cancer Immunome Atlas (https://tcia.at).

## Abbreviations

HCC: Hepatocellular carcinoma
m6A: N6-methyladenosine
TME: Tumor microenvironment
TMB: Tumor mutation burden
TCGA: The Cancer Genome Atlas
GEO: Gene Expression Omnibus
PD-1/PD-L1: Programmed cell death-1 and its ligand
CTLA4: Cytotoxic T lymphocyte associated antigen 4
CNV: Copy number variation
IPS: Immunophenoscore
CDF: Cumulative distribution function
CM: Consistent matrix
PCA: Principal component analysis
GSVA: Gene set variation analysis
ssGSEA: Single-sample gene set enrichment analysis
DEG: Differentially expressed gene
KEGG: Kyoto Encyclopedia of Genes and Genomes
ROC: Receiver operating characteristic
AUC: Areas under the curve

## References

[1] Sung H, Ferlay J, Siegel RL, Laversanne M, Soerjomataram I, Jemal A (2021). Global cancer statistics 2020: GLOBOCAN estimates of incidence and mortality worldwide for 36 cancers in 185 countries. CA Cancer J Clin.

[2] Zhao BS, Roundtree IA, He C (2017). Post-transcriptional gene regulation by mRNA modifications. Nat Rev Mol Cell Biol.

[3] Zaccara S, Ries RJ, Jaffrey SR (2019). Reading, writing and erasing mRNA methylation. Nat Rev Mol Cell Biol.

[4] Chen M, Wong CM (2020). The emerging roles of N6-methyladenosine (m6A) deregulation in liver carcinogenesis. Mol Cancer.

[5] Chen M, Wei L, Law CT, Tsang FH, Shen J, Cheng CL (2018). RNA N6-methyladenosine methyltransferase-like 3 promotes liver cancer progression through YTHDF2-dependent posttranscriptional silencing of SOCS2. Hepatology.

[6] Chen Y, Peng C, Chen J, Chen D, Yang B, He B (2019). WTAP facilitates progression of hepatocellular carcinoma via m6A-HuR-dependent epigenetic silencing of ETS1. Mol Cancer.

[7] Negrini S, Gorgoulis VG, Halazonetis TD (2010). Genomic instability—an evolving hallmark of cancer. Nat Rev Mol Cell Biol.

[8] Alexandrov LB, Kim J, Haradhvala NJ, Huang MN, Tian Ng AW, Wu Y (2020). The repertoire of mutational signatures in human cancer. Nature.

[9] Lee DW, Han SW, Bae JM, Jang H, Han H, Kim H (2019). Tumor mutation burden and prognosis in patients with colorectal cancer treated with adjuvant fluoropyrimidine and oxaliplatin. Clin Cancer Res.

[10] Chan TA, Yarchoan M, Jaffee E, Swanton C, Quezada SA, Stenzinger A (2019). Development of tumor mutation burden as an immunotherapy biomarker: utility for the oncology clinic. Ann Oncol.

[11] Zhang B, Wu Q, Li B, Wang D, Wang L, Zhou YL (2020). m(6)A regulator-mediated methylation modification patterns and tumor microenvironment infiltration characterization in gastric cancer. Mol Cancer.

[12] Hinshaw DC, Shevde LA (2019). The tumor microenvironment innately modulates cancer progression. Cancer Res.

[13] Ribas A, Wolchok JD (2018). Cancer immunotherapy using checkpoint blockade. Science.

[14] Le DT, Kavan P, Kim TW, Burge ME, Cutsem EV, Hara H (2018). KEYNOTE-164: pembrolizumab for patients with advanced microsatellite instability high (MSI-H) colorectal cancer. J Clin Oncol.

[15] Yi L, Wu G, Guo L, Zou X, Huang P (2020). Comprehensive analysis of the PD-L1 and immune infiltrates of m(6)A RNA methylation regulators in head and neck squamous cell carcinoma. Mol Ther Nucleic Acids.

[16] Xu S, Tang L, Dai G, Luo C, Liu Z (2020). Expression of m6A regulators correlated with immune microenvironment predicts therapeutic efficacy and prognosis in gliomas. Front Cell Dev Biol..

[17] Li Y, Gu J, Xu F, Zhu Q, Chen Y, Ge D (2020). Molecular characterization, biological function, tumor microenvironment association and clinical significance of m6A regulators in lung adenocarcinoma. Brief Bioinform.

[18] Yue Y, Liu J, Cui X, Cao J, Luo G, Zhang Z (2018). VIRMA mediates preferential m(6)A mRNA methylation in 3′UTR and near stop codon and associates with alternative polyadenylation. Cell Discov.

[19] Chong W, Shang L, Liu J, Fang Z, Du F, Wu H (2021). m(6)A regulator-based methylation modification patterns characterized by distinct tumor microenvironment immune profiles in colon cancer. Theranostics.

[20] Jiang X, Liu B, Nie Z, Duan L, Xiong Q, Jin Z (2021). The role of m6A modification in the biological functions and diseases. Signal Transduct Target Ther.

[21] Wilkerson MD, Hayes DN (2010). ConsensusClusterPlus: a class discovery tool with confidence assessments and item tracking. Bioinformatics.

[22] Kanehisa M, Goto S (2000). KEGG: kyoto encyclopedia of genes and genomes. Nucleic Acids Res.

[23] Kanehisa M, Furumichi M, Sato Y, Ishiguro-Watanabe M, Tanabe M (2021). KEGG: integrating viruses and cellular organisms. Nucleic Acids Res.

[24] Chen XY, Zhang J, Zhu JS (2019). The role of m(6)A RNA methylation in human cancer. Mol Cancer.

[25] Yang N, Wang T, Li Q, Han F, Wang Z, Zhu R (2021). HBXIP drives metabolic reprogramming in hepatocellular carcinoma cells via METTL3-mediated m6A modification of HIF-1α. J Cell Physiol.

[26] Ma H, Wang X, Cai J, Dai Q, Natchiar SK, Lv R (2019). N(6-)Methyladenosine methyltransferase ZCCHC4 mediates ribosomal RNA methylation. Nat Chem Biol.

[27] Li W, Dai Y, Shi B, Yue F, Zou J, Xu G (2020). LRPPRC sustains Yap-P27-mediated cell ploidy and P62-HDAC6-mediated autophagy maturation and suppresses genome instability and hepatocellular carcinomas. Oncogene.

[28] Yan Y, Huang P, Mao K, He C, Xu Q, Zhang M (2021). Anti-oncogene PTPN13 inactivation by hepatitis B virus X protein counteracts IGF2BP1 to promote hepatocellular carcinoma progression. Oncogene.

[29] Pu J, Wang J, Qin Z, Wang A, Zhang Y, Wu X (2020). IGF2BP2 promotes liver cancer growth through an m6A-FEN1-dependent mechanism. Front Oncol.

[30] Li J, Zhu L, Shi Y, Liu J, Lin L, Chen X (2019). m6A demethylase FTO promotes hepatocellular carcinoma tumorigenesis via mediating PKM2 demethylation. Am J Transl Res.

[31] Li J, Wang W, Zhou Y, Liu L, Zhang G, Guan K (2021). m6A regulator-associated modification patterns and immune infiltration of the tumor microenvironment in hepatocarcinoma. Front Cell Dev Biol..

[32] Zhang X, Shen L, Cai R, Yu X, Yang J, Wu X (2021). Comprehensive analysis of the immune-oncology targets and immune infiltrates of N (6)-methyladenosine-related long noncoding RNA regulators in breast cancer. Front Cell Dev Biol..

[33] Peng H, Zhang Y, Zhou Z, Guo Y, Huang X, Westover KD (2019). Intergrated analysis of ELMO1, serves as a link between tumour mutation burden and epithelial–mesenchymal transition in hepatocellular carcinoma. EBioMedicine.

[34] Huo J, Wu L, Zang Y (2020). A prognostic model of 15 immune-related gene pairs associated with tumor mutation burden for hepatocellular carcinoma. Front Mol Biosci.

[35] Long J, Wang A, Bai Y, Lin J, Yang X, Wang D (2019). Development and validation of a TP53-associated immune prognostic model for hepatocellular carcinoma. EBioMedicine.

[36] Ling S, Shan Q, Zhan Q, Ye Q, Liu P, Xu S (2020). USP22 promotes hypoxia-induced hepatocellular carcinoma stemness by a HIF1α/USP22 positive feedback loop upon TP53 inactivation. Gut.

[37] Yang H, Sun L, Guan A, Yin H, Liu M, Mao X (2021). Unique TP53 neoantigen and the immune microenvironment in long-term survivors of Hepatocellular carcinoma. Cancer Immunol Immunother.

[38] Yuan SX, Wang J, Yang F, Tao QF, Zhang J, Wang LL (2016). Long noncoding RNA DANCR increases stemness features of hepatocellular carcinoma by derepression of CTNNB1. Hepatology.

