# Supplementary material for: Comprehensive characterization of m6A methylation and its impact on prognosis, genome instability, and tumor microenvironment in hepatocellular carcinoma
Source: BMC Med Genomics. 2022 Mar 8;15:53. doi: 10.1186/s12920-022-01207-x (PMC8905789; doi:10.1186/s12920-022-01207-x)
Supplement: Supplementary file 1 — Additional file 1. Fig. S1. PRISMA flow diagram for data collection. Fig. S2. Survival analyses for m6A regulators associated with overall survival of patients with HCC. Fig. S3. Unsupervised consensus clustering analysis of patients with HCC. Fig. S4. GSVA analyses of the three m6A regulator-based expression patterns. Table S1. Prognostic m6A-related genes among three m6A regulator-based expression patterns. [file 12920_2022_1207_MOESM1_ESM.docx]

**Supplementary materials**


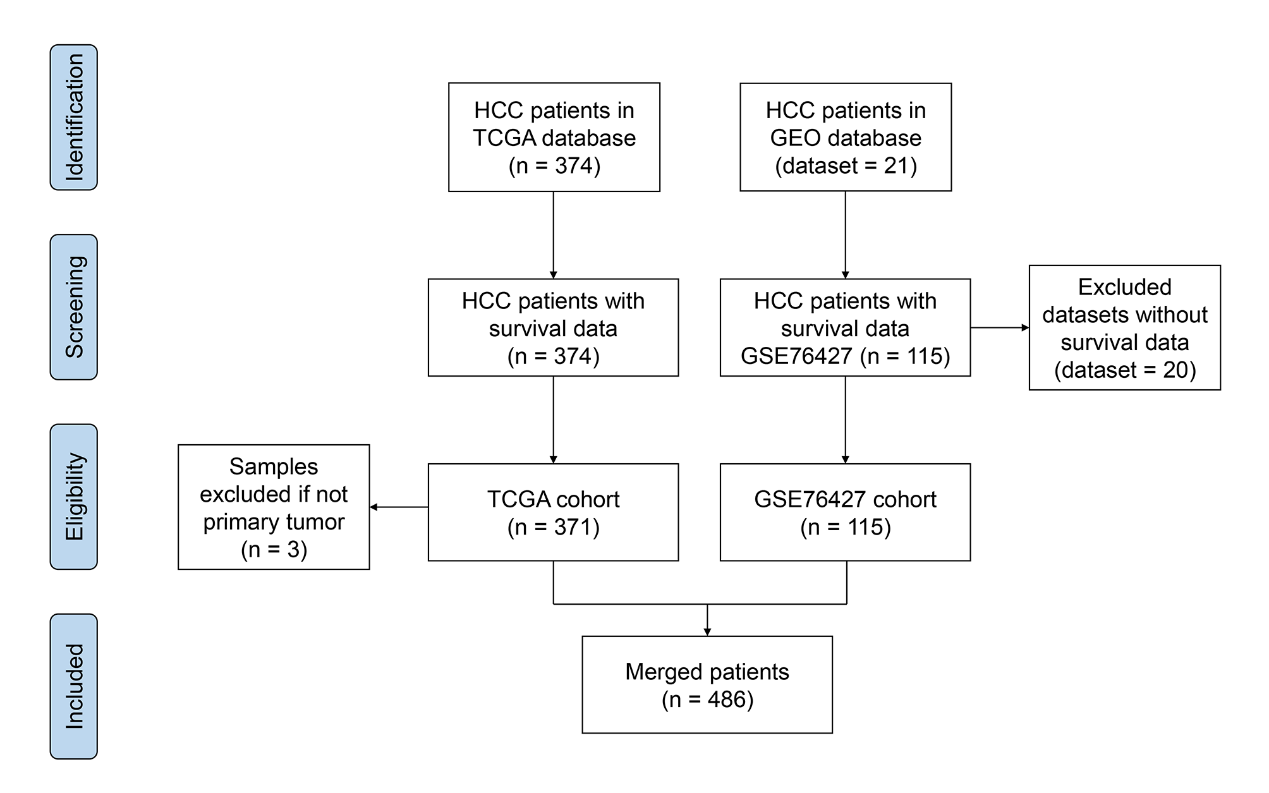


**Fig. S1** PRISMA flow diagram for data collection.


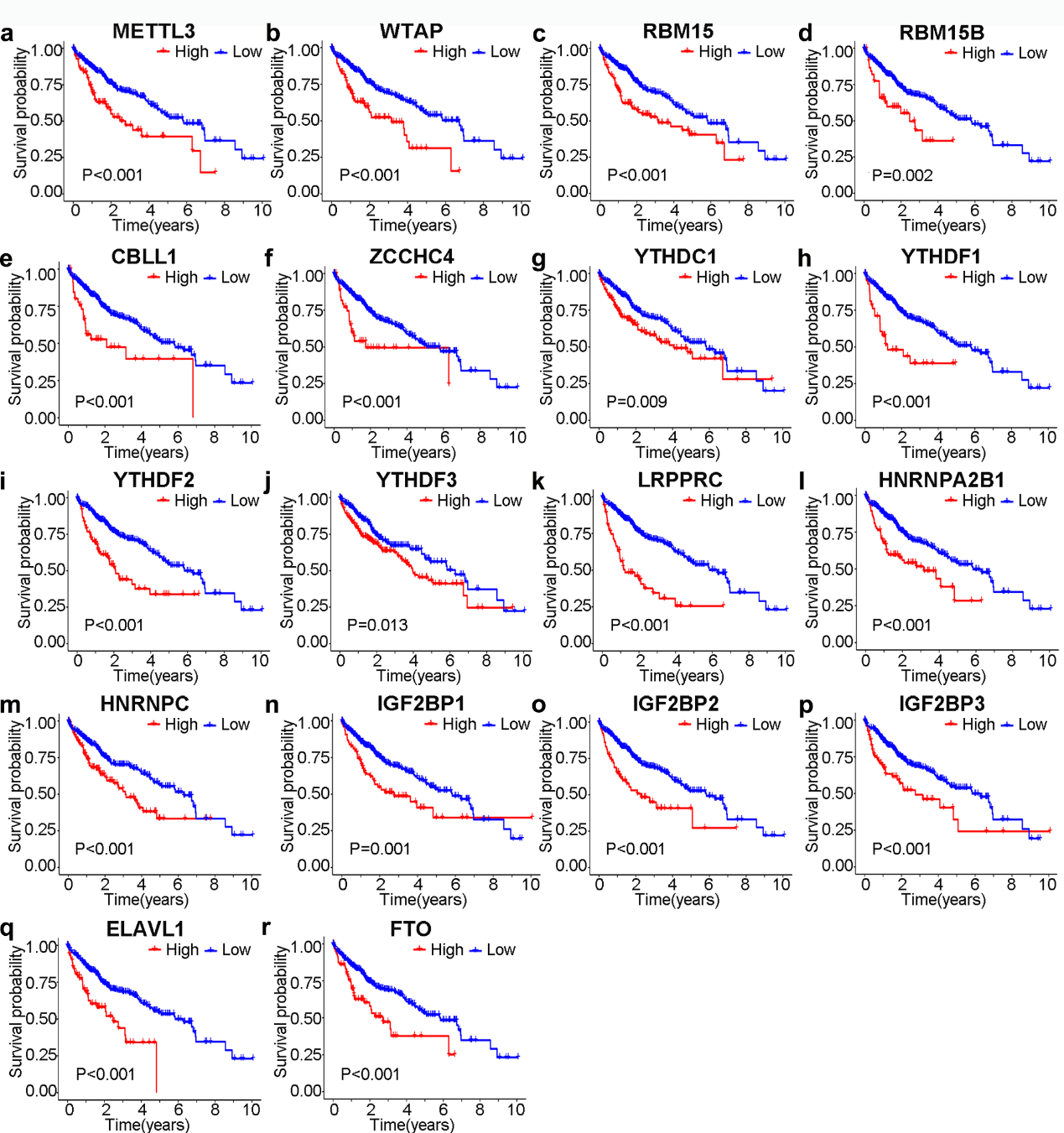


**Fig. S2** Survival analyses for m6A regulators associated with overall survival of patients with HCC. Prognostic m6A regulators from the TCGA and GSE76427 cohorts, including *METTL3* **(a)**, *WTAP* **(b)**, *RBM15* **(c)**, *RBM15B* **(d)**, *CBLL1* **(e)**, *ZCCHC4* **(f)**, *YTHDC1* **(g)**, *YTHDF1* **(h)**, *YTHDF2* **(i)**, *YTHDF3* **(j)**, *LRPPRC* **(k)**, *HNRNPA2B1* **(l)**, *HNRNPC* **(m)**, *IGF2BP1* **(n)**, *IGF2BP2* **(o)**, *IGF2BP3* **(p)**, *ELAVL1* **(q)** and *FTO* **(r)**.


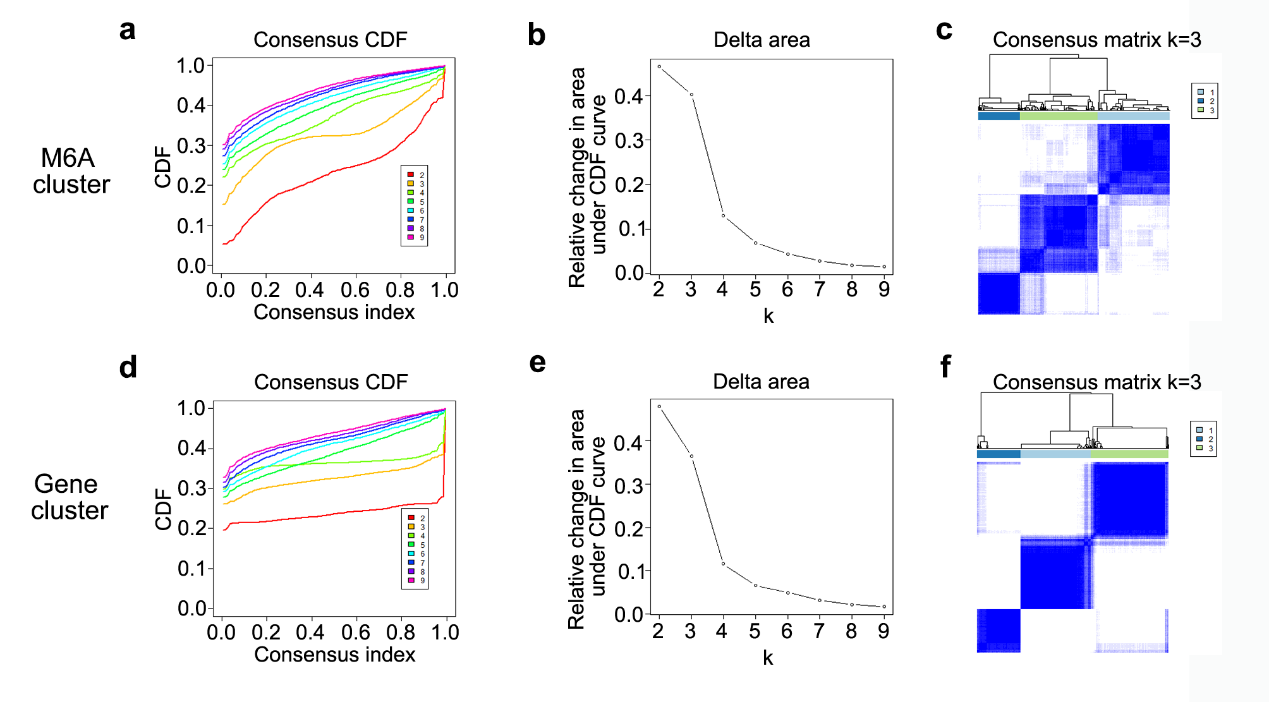


**Fig. S3** Unsupervised consensus clustering analysis of patients with HCC. **a** Empirical cumulative distribution function plots revealed the consensus distributions for each k of m6A clusters. **b** Delta area plot displayed the relative growth in m6A cluster stability. **c** Consensus matrix plot exhibited the m6A clusters at k = 3. The cumulative distribution function plot **(d),** delta area plot (**e**) and consensus matrix plot **(f)** for m6A gene clusters.


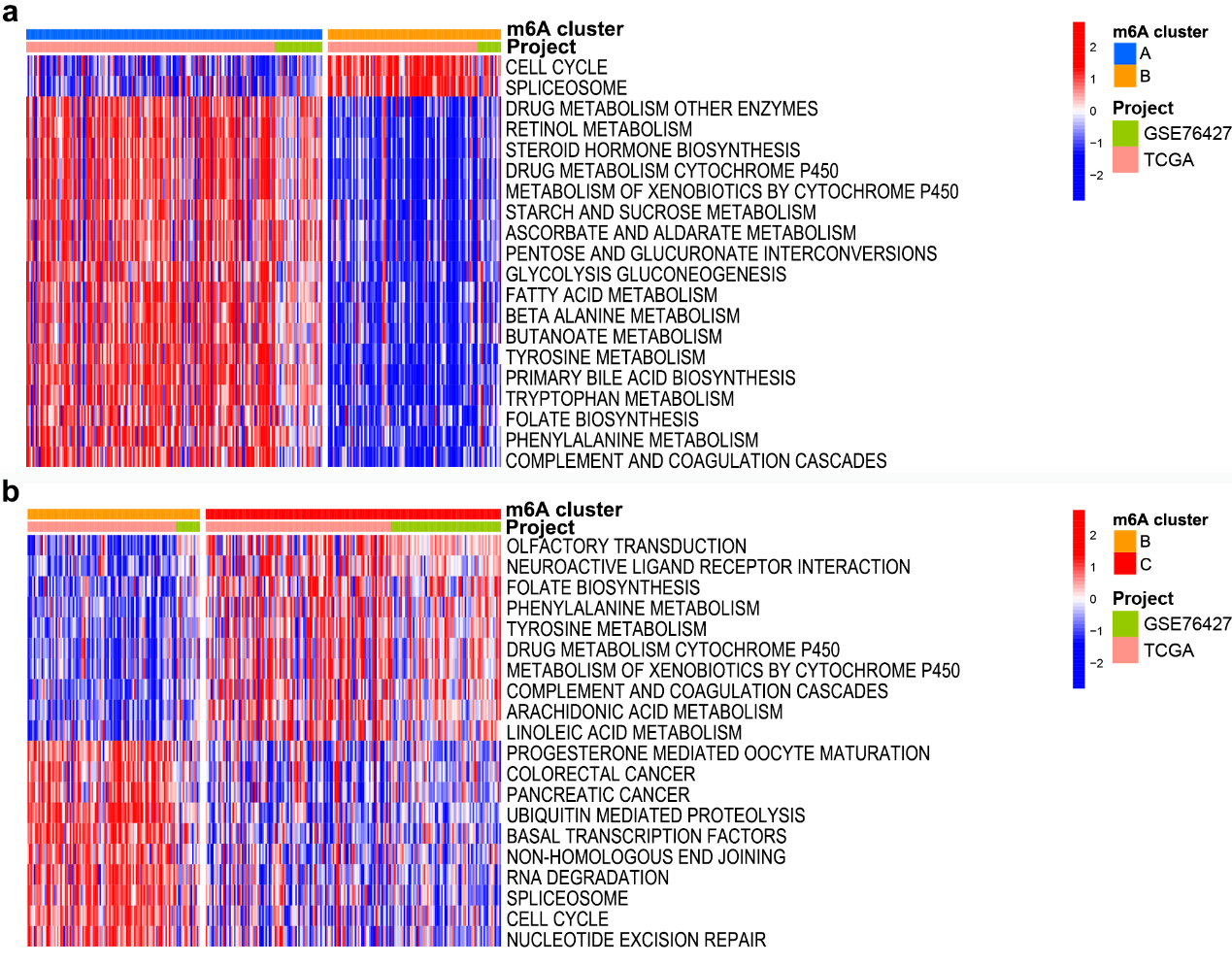


**Fig. S4** GSVA analyses of the three m6A regulator-based expression patterns. Biological pathways activated or inhibited in m6A cluster A vs. cluster B **(a)** and in cluster B vs. cluster C **(b)**.

**Table S1** Prognostic m6A-related genes among three m6A regulator-based expression patterns

| ID | HR | HR.95%L | HR.95%H | *P* value |
| --- | --- | --- | --- | --- |
| *BEND3* | 1.885385 | 1.444088 | 2.461538 | 3.15E-06 |
| *FAM136A* | 1.863413 | 1.427937 | 2.431697 | 4.58E-06 |
| *KIF18B* | 1.444289 | 1.234773 | 1.689356 | 4.28E-06 |
| *DENR* | 1.786302 | 1.349027 | 2.365315 | 5.12E-05 |
| *PLK1* | 1.467327 | 1.267056 | 1.699255 | 3.03E-07 |
| *GAS2L3* | 1.508266 | 1.251967 | 1.817034 | 1.53E-05 |
| *CDC25A* | 1.428255 | 1.200169 | 1.699687 | 5.94E-05 |
| *WASF1* | 1.471323 | 1.22123 | 1.772632 | 4.85E-05 |
| *MYBL2* | 1.25329 | 1.123755 | 1.397758 | 4.99E-05 |
| *CENPO* | 1.507437 | 1.229025 | 1.848918 | 8.17E-05 |
| *TTK* | 1.43975 | 1.223562 | 1.694135 | 1.13E-05 |
| *NAP1L1* | 1.558934 | 1.288048 | 1.88679 | 5.14E-06 |
| *GTSE1* | 1.359896 | 1.16522 | 1.587098 | 9.63E-05 |
| *ANLN* | 1.371131 | 1.194784 | 1.573507 | 7.00E-06 |
| *CPSF6* | 1.895938 | 1.444686 | 2.48814 | 3.98E-06 |
| *DLGAP5* | 1.414874 | 1.2198 | 1.641145 | 4.54E-06 |
| *KIF18A* | 1.648979 | 1.354881 | 2.006916 | 6.03E-07 |
| *SMARCD1* | 1.605084 | 1.268247 | 2.031383 | 8.24E-05 |
| *PRR11* | 1.45987 | 1.245382 | 1.711298 | 3.06E-06 |
| *PRKCD* | 1.474215 | 1.232566 | 1.76324 | 2.15E-05 |
| *GINS1* | 1.462952 | 1.24787 | 1.715107 | 2.74E-06 |
| *NCAPG* | 1.368384 | 1.17982 | 1.587086 | 3.39E-05 |
| *BUB1* | 1.450804 | 1.235678 | 1.703382 | 5.51E-06 |
| *KPNA2* | 1.660962 | 1.397172 | 1.974557 | 8.91E-09 |
| *HJURP* | 1.370305 | 1.178966 | 1.592699 | 4.03E-05 |
| *KIF20A* | 1.394028 | 1.205549 | 1.611973 | 7.39E-06 |
| *OLA1* | 2.136968 | 1.588659 | 2.87452 | 5.17E-07 |
| *NUF2* | 1.353929 | 1.171822 | 1.564337 | 3.93E-05 |
| *CEP55* | 1.432918 | 1.238164 | 1.658305 | 1.39E-06 |
| *CCNB1* | 1.382155 | 1.202106 | 1.589172 | 5.50E-06 |
| *SPC25* | 1.513245 | 1.275204 | 1.79572 | 2.10E-06 |
| *MCM10* | 1.572148 | 1.31228 | 1.883478 | 9.20E-07 |
| *ANAPC7* | 2.075553 | 1.522304 | 2.829867 | 3.90E-06 |
| *MAPKAPK5* | 2.24007 | 1.55834 | 3.220039 | 1.32E-05 |
| *TRIP13* | 1.489214 | 1.285156 | 1.725673 | 1.18E-07 |
| *TPX2* | 1.311073 | 1.152836 | 1.49103 | 3.67E-05 |
| *KIF2C* | 1.386899 | 1.211841 | 1.587245 | 2.02E-06 |
| *SKA1* | 1.370052 | 1.188978 | 1.578703 | 1.34E-05 |
| *CDCA2* | 1.541518 | 1.276763 | 1.861174 | 6.76E-06 |
| *CDCA8* | 1.439885 | 1.249888 | 1.658763 | 4.43E-07 |
| *HDAC2* | 1.903593 | 1.495867 | 2.422453 | 1.65E-07 |
| *CENPE* | 1.591682 | 1.298451 | 1.951134 | 7.68E-06 |
| *WDHD1* | 1.634884 | 1.311139 | 2.038569 | 1.27E-05 |
| *ADSL* | 1.839113 | 1.426126 | 2.371694 | 2.66E-06 |
| *CENPA* | 1.468668 | 1.271573 | 1.696312 | 1.72E-07 |
| *E2F6* | 1.940779 | 1.465486 | 2.570222 | 3.72E-06 |
| *CDCA3* | 1.421417 | 1.19814 | 1.686302 | 5.50E-05 |
| *POLQ* | 1.670361 | 1.294322 | 2.155651 | 8.06E-05 |
| *EZH2* | 1.479615 | 1.237263 | 1.769438 | 1.77E-05 |
| *CDC6* | 1.351525 | 1.16768 | 1.564316 | 5.39E-05 |
| *TPD52L2* | 1.660949 | 1.289727 | 2.13902 | 8.45E-05 |
| *PIGU* | 1.593133 | 1.288181 | 1.970276 | 1.74E-05 |
| *RACGAP1* | 1.413421 | 1.197316 | 1.668531 | 4.37E-05 |
| *ECT2* | 1.338312 | 1.15976 | 1.544354 | 6.65E-05 |
| *STIP1* | 1.631404 | 1.282722 | 2.074867 | 6.62E-05 |
| *DBF4* | 1.88081 | 1.451497 | 2.437101 | 1.77E-06 |
| *NCBP2* | 1.828646 | 1.391193 | 2.403655 | 1.51E-05 |
| *RAN* | 1.759879 | 1.390087 | 2.228044 | 2.64E-06 |
| *SNRPD1* | 1.634724 | 1.303561 | 2.050016 | 2.09E-05 |
| *RRM2* | 1.337652 | 1.168203 | 1.53168 | 2.56E-05 |
| *THOC5* | 1.814417 | 1.386481 | 2.374435 | 1.42E-05 |
| *CHEK1* | 1.53595 | 1.255845 | 1.878529 | 2.95E-05 |
| *BCORL1* | 1.539966 | 1.260183 | 1.881865 | 2.44E-05 |
| *CDK4* | 1.44869 | 1.214452 | 1.728107 | 3.80E-05 |
| *SUV39H2* | 2.034267 | 1.553911 | 2.663113 | 2.38E-07 |
| *CHORDC1* | 1.887267 | 1.464307 | 2.432399 | 9.31E-07 |
| *SAAL1* | 1.739291 | 1.318712 | 2.294006 | 8.90E-05 |
| *ERCC6L* | 1.848192 | 1.441589 | 2.369477 | 1.27E-06 |
| *SEPHS1* | 2.236896 | 1.656548 | 3.02056 | 1.49E-07 |
| *SFPQ* | 1.896615 | 1.455994 | 2.470578 | 2.08E-06 |
| *PBK* | 1.385628 | 1.214962 | 1.580267 | 1.15E-06 |
| *CDC20* | 1.275735 | 1.150305 | 1.414842 | 3.99E-06 |
| *TCOF1* | 1.999708 | 1.525291 | 2.621685 | 5.29E-07 |
| *NDC80* | 1.407662 | 1.199813 | 1.651518 | 2.73E-05 |
| *NUP37* | 1.702628 | 1.316802 | 2.201502 | 4.93E-05 |
| *MELK* | 1.346409 | 1.16733 | 1.55296 | 4.41E-05 |
| *MCM6* | 1.37736 | 1.178613 | 1.609622 | 5.65E-05 |
| *SASS6* | 1.650423 | 1.299727 | 2.095744 | 3.94E-05 |
| *EXO1* | 1.435104 | 1.203542 | 1.711218 | 5.73E-05 |
| *DEPDC1* | 1.476878 | 1.248261 | 1.747365 | 5.51E-06 |
| *TRIM28* | 1.645692 | 1.318141 | 2.054638 | 1.09E-05 |
| *BIRC5* | 1.269026 | 1.126117 | 1.430071 | 9.29E-05 |
| *ZWINT* | 1.329833 | 1.152226 | 1.534817 | 9.73E-05 |
| *SSB* | 1.930288 | 1.478869 | 2.519501 | 1.31E-06 |
| *MTMR2* | 1.691764 | 1.357478 | 2.108371 | 2.85E-06 |
| *CCT6A* | 1.664702 | 1.291732 | 2.145361 | 8.22E-05 |
| *HDAC1* | 1.677722 | 1.315156 | 2.140241 | 3.11E-05 |
| *GTF2H1* | 2.088967 | 1.534508 | 2.843767 | 2.86E-06 |
| *CCT2* | 1.896907 | 1.463502 | 2.458662 | 1.31E-06 |
| *DSCC1* | 1.56514 | 1.265409 | 1.935867 | 3.62E-05 |
| *TRMT6* | 1.771314 | 1.366022 | 2.296855 | 1.61E-05 |
| *HSPA14* | 1.764467 | 1.358305 | 2.292079 | 2.10E-05 |
| *NAP1L4* | 1.945366 | 1.421017 | 2.663198 | 3.29E-05 |
| *KIAA1841* | 2.192766 | 1.628938 | 2.951753 | 2.25E-07 |
| *DHX37* | 1.764178 | 1.336423 | 2.328847 | 6.15E-05 |
| *RALA* | 1.909763 | 1.443502 | 2.526631 | 5.89E-06 |
| *PSRC1* | 1.723245 | 1.430244 | 2.076271 | 1.04E-08 |
| *DYNC1LI1* | 2.281048 | 1.651019 | 3.151495 | 5.73E-07 |
| *TRNP1* | 1.219404 | 1.117138 | 1.331031 | 9.06E-06 |
| *SSRP1* | 1.754537 | 1.349022 | 2.281948 | 2.76E-05 |
| *SPAST* | 1.75257 | 1.325878 | 2.316579 | 8.10E-05 |
| *VPS37C* | 1.725064 | 1.320968 | 2.252777 | 6.22E-05 |
| *SPPL3* | 1.839741 | 1.376706 | 2.458511 | 3.77E-05 |
| *UBE2E1* | 1.814171 | 1.391982 | 2.364409 | 1.05E-05 |
| *GTPBP4* | 1.84272 | 1.438358 | 2.36076 | 1.33E-06 |
| *C18orf54* | 2.09016 | 1.572979 | 2.777385 | 3.71E-07 |
| *MARCKSL1* | 1.333418 | 1.168969 | 1.521001 | 1.83E-05 |
| *PTTG1* | 1.296611 | 1.140927 | 1.473539 | 6.89E-05 |
| *PSMC3IP* | 1.653005 | 1.310246 | 2.085429 | 2.24E-05 |
| *MAD2L1* | 1.481115 | 1.2416 | 1.766835 | 1.27E-05 |
| *EFTUD2* | 1.658832 | 1.288822 | 2.135067 | 8.48E-05 |
| *AACS* | 1.651721 | 1.325926 | 2.057567 | 7.58E-06 |
| *NCL* | 1.888941 | 1.409974 | 2.530612 | 2.02E-05 |
| *CENPL* | 1.561066 | 1.262102 | 1.930849 | 4.02E-05 |
| *NIF3L1* | 1.877623 | 1.380246 | 2.554233 | 6.01E-05 |
| *GPSM2* | 1.622605 | 1.307595 | 2.013503 | 1.11E-05 |
| *CENPH* | 1.591712 | 1.290598 | 1.963079 | 1.40E-05 |
| *FAM72A* | 2.611728 | 1.731516 | 3.939395 | 4.70E-06 |
| *BRIX1* | 1.829799 | 1.416289 | 2.36404 | 3.78E-06 |
| *MTHFD1L* | 1.515628 | 1.243694 | 1.84702 | 3.76E-05 |
| *RBM17* | 1.784448 | 1.374784 | 2.316184 | 1.35E-05 |
| *TUBG1* | 1.607839 | 1.307491 | 1.977181 | 6.76E-06 |
| *ESCO2* | 1.764746 | 1.358553 | 2.292386 | 2.08E-05 |
| *SNHG3* | 1.408937 | 1.187561 | 1.671579 | 8.46E-05 |
| *UCK2* | 1.686349 | 1.373806 | 2.069998 | 5.83E-07 |
| *NOP58* | 1.977696 | 1.495347 | 2.615633 | 1.75E-06 |
| *EXOSC3* | 1.845424 | 1.368309 | 2.488903 | 5.96E-05 |
| *IKBIP* | 1.601812 | 1.289454 | 1.989838 | 2.07E-05 |
| *CKS2* | 1.440134 | 1.223337 | 1.695352 | 1.18E-05 |
| *FARSB* | 2.091593 | 1.54139 | 2.838192 | 2.16E-06 |
| *MEX3A* | 1.523126 | 1.296979 | 1.788704 | 2.88E-07 |
| *RBM45* | 2.699073 | 1.802029 | 4.042664 | 1.46E-06 |
| *FAM72B* | 2.276738 | 1.58058 | 3.279517 | 9.94E-06 |
| *NAT10* | 1.897515 | 1.394079 | 2.582755 | 4.66E-05 |
| *AMD1* | 1.791792 | 1.41206 | 2.273641 | 1.59E-06 |
| *SMS* | 1.852018 | 1.477686 | 2.321177 | 8.83E-08 |
| *DCUN1D5* | 1.872245 | 1.465713 | 2.391533 | 5.14E-07 |
| *RAD54B* | 2.552963 | 1.723055 | 3.782594 | 2.98E-06 |
| *PARD6B* | 1.566083 | 1.274163 | 1.924884 | 2.03E-05 |
| *HMMR* | 1.372598 | 1.185344 | 1.589434 | 2.32E-05 |
| *STMN1* | 1.344183 | 1.161177 | 1.556031 | 7.46E-05 |
| *PLOD2* | 1.437388 | 1.223095 | 1.689226 | 1.06E-05 |
| *ING5* | 2.064934 | 1.453965 | 2.932636 | 5.10E-05 |
| *TUBA1C* | 1.437355 | 1.205706 | 1.71351 | 5.20E-05 |
| *CCT5* | 1.675326 | 1.34872 | 2.081024 | 3.10E-06 |
| *UBE2L3* | 1.82888 | 1.356647 | 2.465491 | 7.45E-05 |
| *FAM72D* | 2.068814 | 1.46904 | 2.913462 | 3.16E-05 |
| *UBE2D2* | 2.004105 | 1.443993 | 2.781479 | 3.23E-05 |
| *PDSS1* | 1.835596 | 1.447022 | 2.328515 | 5.60E-07 |
| *ATIC* | 1.830991 | 1.429138 | 2.345839 | 1.72E-06 |
| *GLRX3* | 1.763178 | 1.335149 | 2.328426 | 6.41E-05 |
| *TPRKB* | 1.894935 | 1.417018 | 2.534038 | 1.63E-05 |
| *HSPD1* | 1.797687 | 1.368177 | 2.362032 | 2.55E-05 |
| *NPM1* | 1.68471 | 1.330785 | 2.132761 | 1.46E-05 |
| *STX3* | 1.401499 | 1.189719 | 1.650978 | 5.38E-05 |
| *MPZL1* | 1.546171 | 1.268755 | 1.884244 | 1.57E-05 |
| *GIT1* | 1.605597 | 1.265246 | 2.037503 | 9.80E-05 |
| *FSD1L* | 2.763435 | 1.871758 | 4.079894 | 3.16E-07 |
| *VPS26A* | 1.888404 | 1.405912 | 2.536481 | 2.41E-05 |
| *POLA2* | 1.550918 | 1.24666 | 1.929432 | 8.19E-05 |
| *STK25* | 1.931354 | 1.404323 | 2.656176 | 5.16E-05 |
| *SMYD5* | 1.756801 | 1.357615 | 2.27336 | 1.83E-05 |
| *PDCD2* | 1.881777 | 1.384749 | 2.557203 | 5.34E-05 |
| *TMEM120B* | 2.096202 | 1.450576 | 3.029184 | 8.14E-05 |
| *CFHR4* | 0.85324 | 0.787795 | 0.924121 | 9.70E-05 |
| *PRR13* | 1.778141 | 1.331463 | 2.37467 | 9.64E-05 |
| *GRPEL2* | 2.122435 | 1.547214 | 2.911512 | 3.07E-06 |
| *ZNF239* | 1.432406 | 1.209138 | 1.696902 | 3.23E-05 |
| *C18orf21* | 1.935251 | 1.411585 | 2.653185 | 4.11E-05 |
| *PFKFB4* | 1.394431 | 1.22063 | 1.592977 | 9.81E-07 |
| *SMOX* | 1.325509 | 1.160856 | 1.513517 | 3.13E-05 |
| *ANP32B* | 1.737134 | 1.348805 | 2.237266 | 1.89E-05 |
| *PRKRA* | 1.982864 | 1.476899 | 2.662167 | 5.26E-06 |
| *PPM1G* | 2.283431 | 1.720868 | 3.029899 | 1.06E-08 |
| *CPSF3* | 2.249144 | 1.618878 | 3.124789 | 1.36E-06 |
| *CAPN10* | 2.053846 | 1.436603 | 2.93629 | 7.93E-05 |
| *POLR2H* | 1.685063 | 1.307355 | 2.171896 | 5.59E-05 |
| *REXO4* | 1.769817 | 1.343698 | 2.331068 | 4.86E-05 |
| *TIMM23* | 2.835425 | 1.9288 | 4.168205 | 1.15E-07 |
| *MRPS23* | 1.713221 | 1.32071 | 2.222384 | 5.01E-05 |
| *SLC1A5* | 1.286282 | 1.154986 | 1.432502 | 4.58E-06 |
| *PUS1* | 1.719486 | 1.312637 | 2.252436 | 8.33E-05 |
| *DHX34* | 1.708233 | 1.329119 | 2.195484 | 2.89E-05 |
| *OTUB1* | 1.932903 | 1.415137 | 2.640109 | 3.43E-05 |
| *EIF1AD* | 2.257951 | 1.533492 | 3.32466 | 3.69E-05 |
| *TSEN34* | 1.852978 | 1.368652 | 2.508693 | 6.60E-05 |
| *RTN3* | 1.803647 | 1.394668 | 2.332557 | 6.94E-06 |
| *NEIL3* | 1.553909 | 1.283588 | 1.881159 | 6.17E-06 |
| *MBOAT7* | 1.709328 | 1.325745 | 2.203895 | 3.55E-05 |
| *CCDC137* | 1.738219 | 1.377851 | 2.19284 | 3.10E-06 |
| *C11orf49* | 1.53174 | 1.239385 | 1.893057 | 7.94E-05 |
| *PRELID2* | 1.915499 | 1.437324 | 2.552757 | 9.17E-06 |
| *CAD* | 1.627026 | 1.317617 | 2.009092 | 6.10E-06 |
| *RANBP1* | 1.701993 | 1.348276 | 2.148506 | 7.68E-06 |
| *GPN1* | 1.912037 | 1.419847 | 2.574844 | 1.97E-05 |
| *DTYMK* | 1.566506 | 1.264284 | 1.940974 | 4.06E-05 |
| *TTC27* | 1.901819 | 1.402358 | 2.579168 | 3.54E-05 |
| *RP9P* | 1.598379 | 1.304067 | 1.959112 | 6.27E-06 |
| *EIF3B* | 1.578192 | 1.260484 | 1.97598 | 6.94E-05 |
| *DCTN2* | 1.784564 | 1.358055 | 2.345023 | 3.24E-05 |
| *MED10* | 1.576915 | 1.254016 | 1.982957 | 9.77E-05 |
| *GPN2* | 1.839252 | 1.355265 | 2.49608 | 9.19E-05 |
| *NOL10* | 2.145711 | 1.595942 | 2.884863 | 4.30E-07 |
| *EIF2B5* | 2.29134 | 1.605846 | 3.269453 | 4.84E-06 |
| *HAVCR1* | 1.340353 | 1.16914 | 1.53664 | 2.66E-05 |
| *KCMF1* | 2.400142 | 1.641976 | 3.508383 | 6.18E-06 |
| *CD3EAP* | 1.675088 | 1.293134 | 2.16986 | 9.35E-05 |
| *FHL3* | 1.503587 | 1.23431 | 1.831609 | 5.11E-05 |
| *UBE2S* | 1.546974 | 1.304884 | 1.833978 | 5.04E-07 |
| *IQGAP3* | 1.423702 | 1.2125 | 1.671693 | 1.62E-05 |
| *MRPL9* | 1.604039 | 1.275008 | 2.01798 | 5.48E-05 |
| *KTI12* | 2.052612 | 1.491682 | 2.824473 | 1.01E-05 |
| *MED8* | 2.212614 | 1.619891 | 3.022217 | 5.98E-07 |
| *SLC39A10* | 1.510421 | 1.252962 | 1.820783 | 1.52E-05 |
| *PSMD14* | 2.023791 | 1.551541 | 2.639783 | 1.99E-07 |
| *CCDC34* | 1.427213 | 1.2026 | 1.693778 | 4.67E-05 |
| *G6PD* | 1.297409 | 1.167571 | 1.441686 | 1.30E-06 |
| *TMEM106C* | 1.411586 | 1.189389 | 1.675293 | 7.99E-05 |
| *DYNLL1* | 1.834965 | 1.364766 | 2.46716 | 5.85E-05 |
| *MRPL53* | 1.806297 | 1.355517 | 2.406986 | 5.42E-05 |
| *GPRIN1* | 1.474793 | 1.217606 | 1.786303 | 7.08E-05 |
| *GNG5* | 1.801694 | 1.379048 | 2.353872 | 1.59E-05 |
| *SUB1* | 1.551936 | 1.244526 | 1.93528 | 9.53E-05 |
| *EEF1E1* | 1.517766 | 1.234781 | 1.865605 | 7.40E-05 |
| *CCT7* | 1.934965 | 1.43009 | 2.618078 | 1.88E-05 |
| *CLCN2* | 1.744641 | 1.370913 | 2.220252 | 6.04E-06 |
| *PSMA1* | 2.180521 | 1.54567 | 3.076123 | 8.99E-06 |
| *IMPDH1* | 1.349611 | 1.17593 | 1.548943 | 1.99E-05 |
| *TXNL4A* | 1.895756 | 1.443233 | 2.490167 | 4.30E-06 |
| *IER3IP1* | 2.065366 | 1.502606 | 2.838893 | 7.86E-06 |
| *CFL1* | 1.876701 | 1.410475 | 2.497037 | 1.56E-05 |
| *CCT4* | 1.990854 | 1.517079 | 2.612586 | 6.85E-07 |
| *ANXA10* | 0.823147 | 0.748041 | 0.905794 | 6.70E-05 |
| *TXNDC9* | 1.788499 | 1.335767 | 2.394676 | 9.46E-05 |
| *RNF7* | 1.989042 | 1.439752 | 2.747895 | 3.04E-05 |
| *SLC38A1* | 1.255479 | 1.12008 | 1.407247 | 9.32E-05 |
| *YKT6* | 1.670514 | 1.309518 | 2.131026 | 3.62E-05 |
| *EXTL2* | 1.739144 | 1.363365 | 2.218498 | 8.37E-06 |
| *YBX1* | 1.924011 | 1.493805 | 2.478114 | 4.02E-07 |
| *LPCAT1* | 1.457878 | 1.264414 | 1.680944 | 2.11E-07 |
| *ACYP1* | 1.653668 | 1.305529 | 2.094644 | 3.04E-05 |
| *ZCCHC17* | 1.741491 | 1.317256 | 2.302356 | 9.85E-05 |
| *AUP1* | 2.113139 | 1.468309 | 3.041155 | 5.63E-05 |
| *FZD7* | 1.394708 | 1.20123 | 1.619348 | 1.26E-05 |
| *EIF3M* | 2.143849 | 1.561803 | 2.942808 | 2.37E-06 |
| *KDELR1* | 1.768303 | 1.334114 | 2.343799 | 7.33E-05 |
| *FTSJ1* | 1.757644 | 1.350036 | 2.288318 | 2.80E-05 |
| *EIF5B* | 2.19918 | 1.625644 | 2.975063 | 3.19E-07 |
| *ZNF408* | 1.949042 | 1.41973 | 2.675695 | 3.66E-05 |
| *BCAT1* | 1.424186 | 1.217005 | 1.666637 | 1.04E-05 |
| *PPIH* | 1.661344 | 1.305895 | 2.113542 | 3.58E-05 |
| *RPF2* | 1.807125 | 1.368025 | 2.387165 | 3.10E-05 |
| *ETV5* | 1.541609 | 1.301996 | 1.825318 | 5.12E-07 |
| *TTLL1* | 1.669467 | 1.293184 | 2.155238 | 8.39E-05 |
| *PTPMT1* | 1.952206 | 1.414575 | 2.694172 | 4.70E-05 |
| *MYCBP* | 1.758819 | 1.343315 | 2.302844 | 4.02E-05 |
| *CCDC58* | 1.845614 | 1.386547 | 2.456671 | 2.67E-05 |
| *TIMM9* | 1.849655 | 1.362307 | 2.511345 | 8.10E-05 |
| *METTL1* | 1.635693 | 1.279848 | 2.090475 | 8.45E-05 |
| *MRTO4* | 1.707773 | 1.332972 | 2.187961 | 2.30E-05 |
| *MED19* | 2.306218 | 1.684659 | 3.157103 | 1.84E-07 |
| *ITGB1BP1* | 1.872609 | 1.451752 | 2.415471 | 1.36E-06 |
| *TRAPPC4* | 1.716913 | 1.327572 | 2.220439 | 3.80E-05 |
| *SLC16A3* | 1.284619 | 1.150019 | 1.434972 | 9.20E-06 |
| *CLEC3B* | 0.730654 | 0.634692 | 0.841126 | 1.25E-05 |
| *ZNF544* | 1.815142 | 1.39083 | 2.368903 | 1.14E-05 |
| *B4GALT2* | 1.906228 | 1.445152 | 2.514409 | 4.97E-06 |
| *PPIA* | 1.906922 | 1.401595 | 2.594438 | 3.97E-05 |
| *PHOSPHO2* | 1.996669 | 1.499339 | 2.658962 | 2.23E-06 |
| *RRP12* | 1.604952 | 1.282972 | 2.007738 | 3.46E-05 |
| *MRPL17* | 1.71481 | 1.316667 | 2.233347 | 6.31E-05 |
| *ATP1B3* | 1.383215 | 1.186832 | 1.612094 | 3.29E-05 |
| *ELOVL1* | 1.776788 | 1.382039 | 2.284288 | 7.32E-06 |
| *TFAP4* | 1.994122 | 1.451642 | 2.739328 | 2.04E-05 |
| *SSR3* | 1.854545 | 1.374283 | 2.502639 | 5.37E-05 |
| *CCNJL* | 1.755924 | 1.426458 | 2.161487 | 1.10E-07 |
| *WDR55* | 1.997667 | 1.43172 | 2.787328 | 4.67E-05 |
| *ZFP41* | 1.695072 | 1.308597 | 2.195687 | 6.41E-05 |
| *RRAGC* | 1.624977 | 1.274191 | 2.072334 | 9.12E-05 |
| *TMEM115* | 2.174176 | 1.506985 | 3.136755 | 3.28E-05 |
| *SEC61A1* | 1.794208 | 1.3771 | 2.337653 | 1.49E-05 |
| *SLC41A3* | 1.798269 | 1.368713 | 2.362637 | 2.51E-05 |
| *RAMP3* | 0.705416 | 0.60878 | 0.817392 | 3.44E-06 |
| *MYCN* | 1.322598 | 1.164038 | 1.502755 | 1.78E-05 |
| *SLC2A1* | 1.404724 | 1.235643 | 1.596941 | 2.06E-07 |
| *POF1B* | 1.291215 | 1.151858 | 1.44743 | 1.15E-05 |
| *SSBP1* | 2.044124 | 1.464484 | 2.853185 | 2.64E-05 |
| *UQCRH* | 1.703359 | 1.350489 | 2.148431 | 6.90E-06 |
| *MMP1* | 1.424113 | 1.253411 | 1.618063 | 5.73E-08 |
| *ANKRD13B* | 1.688555 | 1.359334 | 2.09751 | 2.20E-06 |
| *LYPLA2* | 1.839858 | 1.363283 | 2.483034 | 6.72E-05 |
| *EPO* | 1.218967 | 1.116541 | 1.33079 | 9.80E-06 |
| *NADSYN1* | 1.78913 | 1.340364 | 2.388145 | 7.88E-05 |
| *GRIN2D* | 1.686539 | 1.303949 | 2.181385 | 6.84E-05 |
| *YIF1B* | 1.60764 | 1.274572 | 2.027744 | 6.12E-05 |
| *RGS20* | 2.549827 | 1.69432 | 3.837301 | 7.18E-06 |
| *RIMS3* | 1.758826 | 1.334892 | 2.317394 | 6.00E-05 |
| *FIBP* | 1.965764 | 1.426161 | 2.709532 | 3.66E-05 |
| *GPATCH4* | 1.57641 | 1.255289 | 1.97968 | 8.99E-05 |
| *FABP6* | 1.364544 | 1.182754 | 1.574276 | 2.04E-05 |
| *CRIPT* | 1.908273 | 1.39685 | 2.606941 | 4.91E-05 |
| *WDR92* | 3.488988 | 1.953131 | 6.232576 | 2.43E-05 |
| *ALDOA* | 1.33211 | 1.154227 | 1.537406 | 8.81E-05 |
| *UBE2F* | 2.155224 | 1.476507 | 3.145931 | 6.91E-05 |
| *COMMD3* | 2.219045 | 1.604247 | 3.069453 | 1.47E-06 |
| *WDR35* | 1.772155 | 1.358705 | 2.311415 | 2.43E-05 |
| *CHAC2* | 1.846074 | 1.401111 | 2.432347 | 1.32E-05 |

*HR: Hazard ratio; L: low; H: high.*
